# The complete chloroplast genome of *Acer pentaphyllum* (Sapindaceae), a critically endangered maple endemic to China

**DOI:** 10.1080/23802359.2019.1704647

**Published:** 2020-01-10

**Authors:** Wentai Dai, Shiqi Li, Xinfen Gao, Bo Xu

**Affiliations:** a Chengdu Institute of Biology, Chinese Academy of Sciences, Chengdu, PR China;; b College of Life Sciences, University of Chinese Academy of Sciences, Beijing, PR China

**Keywords:** *Acer pentaphyllum*, chloroplast genome, phylogenetic analysis

## Abstract

*Acer pentaphyllum* is a critically endangered maple confined to the valley of Yalong River in Southwest China. The whole chloroplast genome of *A. pentaphyllum* was 156,862 bp in length with a typical quadripartite structure, containing a large single-copy (LSC) region of 85,292 bp, a small single-copy (SSC) region of 18,146 bp, and a pair of inverted repeat (IR) regions of 26,712 bp separated among them. Totally 137 unique genes were predicted, comprising 89 protein-coding genes, 39 tRNA genes, and 8 rRNA genes (4 rRNA types). The phylogenetic analysis showed that *A. pentaphyllum* was resolved as sister to the clade containing *A. griseum* and *A. sino-oblongum.* This study reported the first complete chloroplast genome sequences of *A. pentaphyllum* and reconstructed a phylogeny tree based on 16 Sapindaceae species, which may provide new insight into phylogenetic studies of Sapindaceae and further conservation strategies for *A. pentaphyllum*.

*Acer* L. belongs to the subfamily Hippocastanoideae of Sapindaceae (APG 2016). *Acer pentaphyllum* Diels is significantly distinct from other maples, since its leaves with red petioles which were divided to the base into five or occasionally seven narrow leaflets (McNamara 2011). It is the unique species of *Acer* sect. *pentaphylla* Hu & W. C. Cheng, distributed at altitudes of 2300–2900 m (Xu et al. 2008). This species is endemic to Southwest China and assessed as Critically Endangered in the IUCN Red List (Barstow et al. 2019). It is confined to the valley of Yalong River in Sichuan (McNamara 2011, 2017). In this study, we first reported the complete chloroplast genome of *A. pentaphyllum*, which will offer a useful resource for future conservation genetics.

Fresh leaves were collected from a single individual of *A. pentaphyllum* in Liangshan City, Sichuan Province, Southwest China. Its voucher specimen (CDBI: xubo1519) was deposited at the Herbarium of Chengdu Institute of Biology. High-quality total genomic DNA was extracted from dry leaves using a modified CTAB DNA extraction method (Doyle and Doyle 1987) and sequenced by Illumina pair-end technology. The filtered high-quality reads were assembled using the program NOVOPlasty (Dierckxsens et al. 2017), utilizing *Acer catalpifolium* as the reference genome (GenBank accession no. NC041080). The assembled chloroplast genome was annotated using Plann version 1.1 (Huang and Cronk 2015), and the annotation result was modified using Geneious version 10.2.3 (Kearse et al. 2012). The sequences were aligned with the MAFFT (Katoh and Standley 2013). Poorly aligned regions were trimmed using Gblocks version 0.91b (Castresana 2000) with the option ‘-t = c’ (the type of sequence was set to codons). The maximum-likelihood (ML) tree was constructed using RAxML version 8.2.11 (Stamatakis 2006) with 1000 bootstrap replicates to examine the phylogenetic position of *A. pentaphyllum* in genus *Acer*.

The complete chloroplast genome of *A. pentaphyllum* (Genbank accession no. MN661390) is a typical circular double-stranded DNA with a quadripartite structure. It is 156,862 bp in length and consists of the large single-copy (LSC) region of 85,292 bp, small single-copy (SSC) region of 18,146 bp, and two inverted repeat (IR) regions of 26,712 bp. The chloroplast genome had an overall GC content of 37.9%. The chloroplast genome encoded 137 unique genes: 89 protein-coding genes, 39 tRNA genes, and 8 rRNA genes (4 rRNA types). Most of the genes were located in the single-copy regions, while 20 gene species were duplicated in the IR regions. The phylogenetic tree (Figure 1) showed that *A. pentaphyllum* was sister to the clade consisted of *A. griseum* and *A. sino-oblongum*. Moreover, phylogenetic analyses indicated that *A. sino-oblongum* belonging to *A*. sect. *Palmata* was more closely related to *A.* sect. *trifoliata* and *A.* sect. *pentaphylla*, rather than forming a clade with other species in *A*. sect. *Palmata*. This phylogenetic analysis suggested an inconsistent result with previous taxonomic researches (Xu et al. 2008). In order to better resolve this issue, furthermore comprehensive studies are necessary to be conducted by sampling more taxa and combining mutiple evidences.

**Figure 1.**
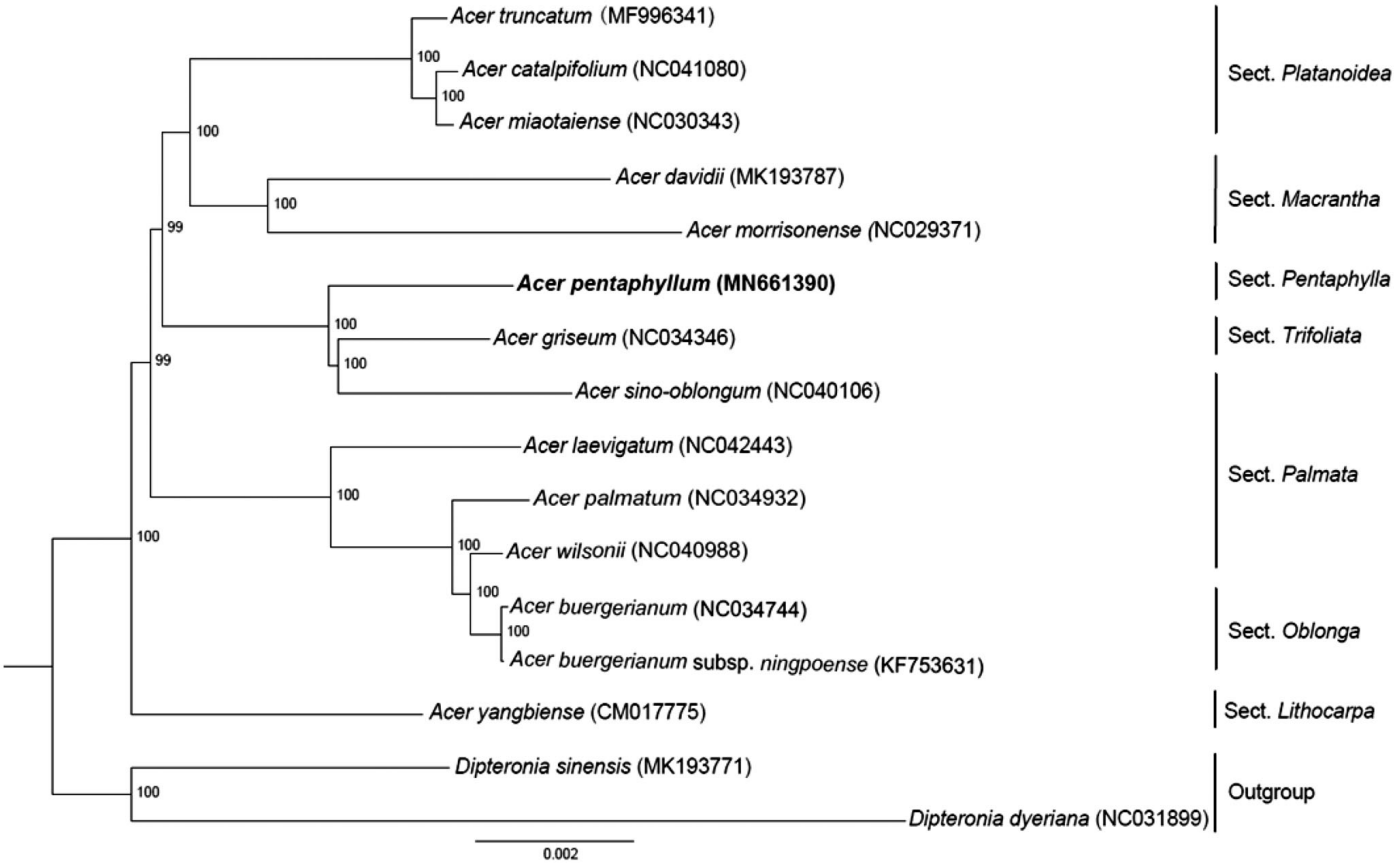
Phylogeny of chloroplast (cp) genome dataset, maximum-likelihood bootstrap support values are shown along the branches.
